# Barriers and facilitators of clinician and researcher collaborations: a qualitative study

**DOI:** 10.1186/s12913-020-05978-w

**Published:** 2020-12-05

**Authors:** Julie Williams, Tom J. Craig, Debbie Robson

**Affiliations:** 1 Health Service and Population Research Department, Institute of Psychiatry, Psychology & Neuroscience, King’s College London, London, UK; 2 Addictions Department, Institute of Psychiatry, Psychology & Neuroscience, King’s College London, London, UK

**Keywords:** Qualitative, Research to practice, Research partnerships, Mental health

## Abstract

**Background:**

The poor translation of research findings into routine clinical practice is common in all areas of healthcare. Having a better understanding of how researchers and clinicians experience engagement in and with research, their working relationships and expectations of each other, may be one way to help to facilitate collaborative partnerships and therefore increase successful translation of research into clinical practice.

**Aims:**

To explore the views of clinical and research staff about their experiences of working together during research projects and identify the facilitators and barriers.

**Methods:**

We conducted four focus groups with 18 participants - clinicians, researchers and those with a dual clinical-research role, recruited from one mental health Trust and one university. Data was analysed using thematic analysis.

**Results:**

Eight themes were identified under the headings of two research questions 1) Barriers and facilitators of either engaging in or with research from the perspective of clinical staff**,** with themes of understanding the benefits of the research; perceived knowledge and personal qualities of researchers; lack of time and organisational support to be involved in and implement research; and lack of feedback about progress and outcome of research. 2) Barriers and facilitators for engaging with clinicians when conducting research, from the perspective of researchers, with themes of understanding what clinicians need to know and how they need to feel to engage with research; demonstrating an understanding of the clinician’s world; navigating through the clinical world; and demands of the researcher role.

**Conclusion:**

There was agreement between clinicians and researchers about the barriers and facilitators for engaging clinicians in research. Both groups identified that it was the researcher’s responsibility to form and maintain good working relationships. Better support for researchers in their role calls for training in communication skills and bespoke training to understand the local context in which research is taking place.

**Supplementary Information:**

The online version contains supplementary material available at 10.1186/s12913-020-05978-w.

## Background

The poor translation of research findings into routine clinical practice is common in all areas of healthcare, including mental health care. A review by Cooksey [1] identified two principal gaps in health research being translated into improvements in clinical practice [1]. The first gap concerns the translation of basic research ideas into the development of new health technologies and health improvement approaches; the second relates to implementing new technologies and approaches into clinical practice. New models of research and clinical practice partnerships have been developed in recent years to facilitate the engagement of clinicians, services users, researchers and policy makers in mutually beneficial programmes of implementation research to bridge the second translational gap, e.g. [2, 3]. In the UK, the National Institute of Health Research (NIHR) was established in 2006 to improve funding and implementation of research in the National Health Service (NHS); one of its initiatives, Collaborations for Leadership in Applied Health Research and Care (CLAHRC) was set up in 2008 to address the second translational gap. CLAHRCs were collaborations between local providers of NHS services, commissioners, universities, and Academic Health Science Networks, designed to promote better integration of research and healthcare systems. Their primary focus was on applied research to improve chronic disease and public health interventions that were generalisable and applicable across the NHS [4]. In 2019, CLAHRCs transformed into Applied Research Collaborations (ARCs), and have been awarded further funding to undertake research to address multimorbidity and an aging society [5]. Early evaluations of CLAHRCs identified collaborative action, relationship building, engagement and knowledge exchange as the key process mechanisms, but found that the academic-clinical practice divide remained evident, with activities being largely investigator-driven, with little impact on health services and outcomes [6]. Other evaluations suggest that CLAHRCs have created opportunities for academics, clinicians and service users to co-produce the research agenda and generate new knowledge, whilst taking into account organisational and geographical contexts in which the research is to be applied [7]. There are many examples of how CLAHRCs have successfully addressed the second translation gap. These include improved health and social outcomes for people with first episode psychosis and widespread adoption of palliative and end of life care outcome measures into routine practice locally, nationally and internationally [8]. The processes that led to these improved health outcomes, however, are less well described.

Collaborative approaches to co-produce research and generate new knowledge are known by various terms such as integrated knowledge translation, participatory, action or community-based research. Such approaches can lead to improved performance within health organisation and improved health outcomes [9]. However, findings from several reviews suggest the mechanisms involved in these approaches are often ill defined [10, 11] and impeded by several barriers such as clinician’s perceived lack time, differing values, group and personal dynamics of all stakeholders involved [12]. Enablers of co-produced research and the generation of new knowledge include, among other things, understanding the differing needs and priorities of researchers, clinicians, and other stakeholders to ensure outcomes meet everyone’s needs [13], having clear expectations between the different collaborators [12] and developing capacity among team members to understand each other’s roles and expectations [14].

Within mental health settings, previous research has focused on barriers to the successful engagement and involvement of clinical staff in the research process, which is necessary to support researchers to recruit and retain service users in clinical studies [15] and a key factor in successfully implementing evidence-based interventions and training [16, 17]. Studies suggest clinician engagement in research is influenced by factors including lack of knowledge of the research process, concern about how involvement in research may affect their relationships with services users, and adding to already demanding workloads [15, 17, 18]. Boaz et al. [10] define engagement ‘in research’ as clinicians playing an active role in the whole research cycle and organisations taking an active role in research networks, collaborations and ensuring the research functions are fully integrated into organisational structures. Whereas engagement ‘with research’ is defined as a less substantial involvement at an individual and team level and relates to receiving and disseminating research findings. Clinical engagement *with* rather than *in* research has a more well-established literature [10].

Although there a multiple ways to close the second research translational gap, having a better understanding of how researchers and clinicians experience engagement in and with research, their working relationships and expectations of each other, may be one way to help to facilitate collaborative partnerships and therefore increase successful translation of research into clinical practice.

### Purpose of the study

We aimed to explore the views of clinical and research staff about their experience of working together on research projects within one NHS Trust and one university with close links.

Our research questions were: 1. What are the barriers and facilitators of either engaging in or with research from the perspective of clinical staff. 2: What are the barriers and facilitators for engaging with clinicians when conducting research, from the perspective of researchers.

### Study context

We conducted the study as part of a programme of applied research within NIHR CLAHRC/ARC South London. At the time of the study the authors worked in the ‘Psychosis Theme’ which aimed to help people who experience psychosis improve their physical health, by increasing access and uptake of evidence-based interventions to improve outcomes related to sedentary behaviours [19, 20] and tobacco smoking [21, 22]. Also integral to this work, we explored staff and organisational barriers to implementing tobacco dependence treatment interventions in mental health settings [21–24]. In preparation for future implementation work, we wanted to understand the barriers and facilitators to engaging in and with research, from the perspectives of clinicians and researchers. We were interested in how they worked together to implement research and evidence-based interventions into clinical practice to both inform our own practice locally and to inform the literature more widely. The study was conducted with clinicians in the South London and Maudsley NHS Foundation Trust (SLaM), a large mental health Trust, and researchers based at the Institute of Psychiatry, Psychology and Neuroscience (IoPPN) which is part of King’s College London (KCL), England. SLaM and the IoPPN are part of King’s Health Partners, an Academic Health Sciences Centre and also part of the South London CLAHRC/ARC. One author worked in SLaM and the IoPPN simultaneously, one worked in SLaM prior to working in IOPPN and one worked at the IoPPN but not in SLaM.

## Methods

We undertook focus groups with clinicians, researchers and staff with a dual clinical/research role. Focus groups were used as they provide a space for participants to discuss and expand on their ideas and experiences with others who have had a similar experience [25, 26]. We developed an interview guide for the study based on our own experiences as clinicians and researchers and the literature available on the engagement of clinical staff in research in mental health settings. The study was designed to explore the views of clinicians and researchers on working with each other in research projects. As such, we were not looking to generate a theory but to provide exploratory data for better understanding this relationship. As we were exploring views in one particular context (in one NHS Mental Health Trust and one University with close links),we were not aiming for data saturation but an exploratory understanding which could inform further local initiatives with the CLARHC/ARC about how to support clinicians and researchers in co-producing and generating new knowledge. We did not aim for a specific sample size due to this being an exploratory study.

### Researcher characteristics and reflexivity

The study was designed, and the data collected and analysed by two of authors (JW & DR), who we are post-doctoral researchers with NIHR CLAHRC/ARC South London. Both worked clinically for many years (JW as an Occupational Therapist and DR as a mental health nurse) before moving to research posts and gaining PhDs, and still have strong links with the clinical world. TJC is an Emeritus professor of social psychiatry. We have all experienced the research process from both the clinical and research perspective and therefore brought this experience and knowledge to design and interpretation of the data. All researchers are white and the two researchers who ran the focus groups are female.

### Participant selection and setting

We emailed managers of services working with people with a diagnosis of psychosis in SLaM and selected heads of departments in the IoPPN, KCL. We purposively recruited participants based on their role as clinicians, researchers and those with a dual role, as well as their work setting. This was to ensure we included a variety of staff working in community and inpatient settings with people with a diagnosis of psychosis and/or had experience of research in this field. For clinician recruitment we did not purposively select participants based on any other characteristic than working in a mental health setting with people with psychosis. For researchers, we wanted to recruit people who were closely involved in working directly with clinicians. We also approached potential participants face to face in clinical teams and at research forums. Potential participants who expressed an interest were then contacted by one of the researchers who give them more information on the study. Some but not all of the participants had a prior relationship with at least one of the researchers due to working alongside them in similar clinical environments.

### Data collection

We undertook four focus groups comprising 1) clinicians working in community mental health services; 2) clinicians working in inpatient mental health services; 3) research staff working in an academic setting and 4) staff who had a dual clinical/research role. The focus groups took place on the university campus and lasted 60–90 min and were facilitated by two of the authors (JW and DR) following an interview guide (see Additional file 1) developed for the study. No-one apart from the researchers and participants were present. Each focus group started with the researchers outlining why they were doing this research and inviting participants to ask any questions they may have. Clinicians were invited to focus their discussions on their experience of working with researchers in the context of any current or past research project and to give examples of good and not so good working relationships. Researchers were asked to do the same with respect to clinicians. Participants with a dual role were asked to discuss their experience of relationships with clinicians. Member checking was conducted during the process of data collection to check data between participants (Morse, 2015). After the first focus group with clinicians, we checked out some of their experiences with the researcher focus group, and for the group with people with a dual role, we checked some of the experiences of the two previous focus groups with them. The focus groups were audiotaped and transcribed by one of the authors (JW). As mentioned above, we did not seek to achieve data saturation in this study as the setting was context specific and this was an exploratory study only.

### Data analysis

The data were analysed by JW and DR after all the focus groups were completed. Thematic analysis was used following the steps outlined by Braun and Clark [14]; (i) we familiarised ourselves with the data by reading the transcripts several times, (ii) we independently generated initial codes, searched for and defined themes; (iii) together we reviewed, described and interpreted an agreed final set of themes. All analysis was done using Microsoft Word. We analysed the data deductively, based on our two research questions. We also used an inductive approach as there were few studies to draw on that have explored the perspectives of both clinician and researchers about working together and these focus on one specific aspect of research, e.g. recruitment [18, 27]. We therefore undertook our analysis without imposing a coding framework on the data. We did this as we were interested in understanding the experiences of participants and identifying the main issues that participants reported on. We did not return transcriptions to participants to check.

### Ethics

Ethical approval was obtained from King’s College London (LRS14/150477). Following the initial approach, potential participants were provided with written and verbal information about the study. This information made it clear that participation was voluntary, and participants could withdraw at any time. Participants gave informed consent to participate. Each participant was given an ID number and all identifiable data were kept in a locked cabinet/password protected computer and only accessible by the research team. All participants were given a £25 voucher to thank them for their time.

## Results

### Participants

Eighteen staff participated in the focus groups. One participant in the clinical focus group dropped out on the day due to work issues. The clinical staff participants comprised of Occupational Therapists, Mental Health Nurses and Clinical Psychologists working in community teams and inpatient settings. The research staff participants and those in a dual clinical/research role included PhD students, research assistants, mental health nurses, midwives and allied health professionals. Fifteen participates were female, the mean age of participants was 35.5 years of age and they had worked in their respective roles for a mean of 3.5 years. The clinical staff had been qualified for a mean of 11.7 years.

### Themes

We identified eight themes in line with the two main research questions.

#### A] Barriers and facilitators of either engaging in or with research from the perspective of clinical staff

Clinicians identified that their perception of the relevance and potential benefit of the research topic to them and the service users they worked with, and the interpersonal and communication skills of researchers positively and negatively influenced their decisions to engage with and implement research. Whereas concern for service users’ welfare, their workload, research fatigue and lack of organisational structures to support research were barriers.

##### Theme A1: Understanding the benefits of the research (*facilitator*)

Clinicians reported that they were more likely to engage with a research project if they understood the benefits of the research and it was personally meaningful and helpful to them and the service users they worked with. They described feeling frustrated if the potential benefits were not clearly communicated by researchers.

*‘I’ve directed patients to research projects within the Trust, which has kind of helped with my side of things because they’d also do other things like test blood pressure, test for other bloods and things like that, so it does help with working collaboratively I think’ (Clinician ID16).*

*‘If they make the people in the team see the relevance it will have to the clinician but also to their clients and also to the service at all the different levels I feel then they are more warmly responded to’ (Clinician ID11).*

They disengaged if they perceived the research project to be of no value to the people they work with and particularly if they perceived that the research study was for the researcher’s purpose of career advancement.

‘*The patients are a very captive audience … I didn’t want to deny them the opportunity of having a bit of money (as patients are paid to take part in research projects) but at the same time, I felt like they were being used and it just never really seemed clear what the projects… how in the end was going to help them… And that happened over and over and over again and sometime the projects were just because somebody wanted to get their own personal qualification’ (Clinician, ID17).*

##### Theme A2: Perceived knowledge and personal qualities of researchers (*facilitator*)

Clinicians identified key engagement skills and qualities that they wanted researchers to possess. The ability to present the research in an appealing way and demonstrating a personal belief in the research project were important. They also suggested that researchers could benefit from training on how to present their research*.*

*‘They need to be quite like charismatic and quite bubbly. If you’re basically trying to sell something, you’ve got to be a good salesperson, have a good sales pitch*’ *(Clinician ID16).*

*‘Maybe researchers need to do some training on marketing and presentation cos I think it is a huge element, like non-verbal body language…….’ (Clinician ID11).*

Clinicians wanted researchers to understand their world. This included demonstrating empathy about the pressures on their time and to have knowledge about the client group their team worked with. There was also some agreement that researchers with a clinical background understood this better.

*‘There is something about a lot of the researchers not having an understanding of our services or what our services do or our client and that brings its own frustration’ (Clinician ID10).*

*‘Researchers that have clinical backgrounds and have been doing what I call work on the ground, they tend to be much more empathic and better communicators and more involved and easier for the teams to work with.’ (Clinician ID12).*

*‘If you have been working clinically you have an understanding why other things come up and then instead of having a quite a short email saying you said you were going to send these names…..Our time is quite precious, if you have got somebody sat downstairs kicking off in reception are you really going to prioritise sending a list of names over and there is that lack of understanding with some researchers.’ (Clinician ID10).*

Clinicians believed it was also important that researchers had an understanding and appreciation of the clinical environment they were working, understand the risks that may arise from working in certain environments and try to fit in.

*“They’ve also got to be reliable, you know, if they say that they’re going to be there at a certain time and with a certain bit of information, whether it’s a leaflet or whatever it is they’ve promised to bring and I expect them to be there and have it with them……. I think it was always important that the people who came to research were able to think about their own personal safety and other people’s safety, it was a big thing for us. So, you know, being able to manage themselves in the environment was so important.’* (*Clinician ID17).*

##### Theme A3: Lack of time and organisational support to be involved in and implement research (*barrier)*

Clinicians reported there was an expectation from their organisation to be involved in research and expressed frustration at the lack of protected time to do this. They also felt research projects were imposed on them and that they were excluded from the early stages of the research process.

*‘If we’re wanting to have a closer relationship with research I really do think there needs to be thought about having protected time for clinicians to implement research …I think there are lots of staff who, even though they might come across quite negative about research, I actually think if they had the chance to participate in something different to their day job they would enjoy it’* (*Clinician ID10).*

*‘No one asks you what you think would be a good idea. Somebody comes along and tells you what is a good idea’. (Clinician ID18*).

##### Theme A4: Lack of feedback about progress and outcome of research *(barrier)*

Clinicians reported they felt researchers worked hard to engage them and communicated frequently when studies were being set up and they needed to recruit participants. However, they reported that this level of engagement and communication did not continue throughout a research project and clinicians were left wondering what happened as a result of the research.

*‘I think something else that can get in the way of the implementation is if the outcome wasn’t what the researchers wanted, if it wasn’t positive, then they’re not going to kind of tell everyone. So even if there was no change, they still don’t let everyone know and the reasons why’. (Clinician ID16).*

*‘I think people would respect it if they got some feedback, even if it was, well you know, we’re not going to change anything as a result of this….I think they’d be like oh ok and they’d accept that…… the idea that you do something and then you never hear about it again, even though in the information it says that you’re going to get the feedback, doesn’t it?...I don’t think I remember seeing any feedback from any projects*. (*Clinician ID18).*

There was clear agreement across all these themes among clinicians from inpatient and community settings. Overall, the issues raised impacted on their willingness to be involved in research and their perception of researchers. Negative experiences of being involved in research in the past were raised by all participants and coloured their views on taking part in any research in the future.

#### B] Barriers and facilitators for engaging with clinicians when conducting research, from the perspective of researchers

All of the researchers perceived that the initiation of a relationship with clinicians was their responsibility and important for the future success of a research project. They demonstrated an understanding of the demands of the clinical world and perceived it was their responsibility to engender interest and engage clinicians. They appreciated that clinicians may have had previous negative experiences of being involved in research and worked hard to mitigate this. A lack of formal training meant they often had to ‘learn on the job’ and feel their way with clinical teams, working out as quickly as possible who were allies and who may impede their job role (e.g. reach a recruitment target).

##### Theme B5: Understanding what clinicians need to know and how they need to feel to engage with research (*facilitator*)

Researchers recognised they needed to work hard to ‘sell’ the research project to clinicians and help them understand the potential benefits during their first meeting. They were also aware that they had to pre-empt concerns about potential risks of the research project and provide clinicians with reassurance.

*‘It also kind of depends on how well what you’re selling gels with what that particular team wants. It’s not just that you’re doing research for research sake. You’re actually going in there to try and improve the sort of the care that these people get given.’* (*Researcher ID 3).*

*‘We start off with the potential benefits, the experiments and the history and how exciting this is and what we might find, but clinicians… maybe the first thing they need to hear, so they can relax and hear what you said after that, is that the risks to your clients, this is tiny.’* (*Researcher ID6).*

*‘There is mileage in just reminding people that research studies that we do, you know, have undergone regulatory ethics approval process and so it’s been scrutinised at so many levels before we come to you as clinicians. And so, you know, that should alleviate some of the anxiety.’ (Researcher ID1).*

##### Theme B6: Demonstrating an understanding of the clinician’s world (*facilitator*)

Researcher participants were very aware and sympathetic of the pressures that clinicians were under and how this could impact on their ability to engage in research activities.

‘*You tend to find with the one’s that aren’t really interested that they probably find researchers a bit of a nuisance. I guess especially if they have heavy caseloads and they have other targets*.’ (*Researcher ID5).*

‘*I’ve kind of felt myself in a team meeting, you know, saying this has the potential to really help with blah, blah, blah and thinking actually this isn’t something you’re going to care about today, this week, in your team meeting, when you know that your most difficult client is about to come in in 20 minutes.’* (*Researcher ID6).*

Researchers experienced a range of reactions from clinicians and discussed different ways of demonstrating to them that they understood the pressures they faced. They often felt they were interrupting clinical work and tried to be mindful of their competing demands.

*‘I think the respect element, definitely. I try to, whenever meeting with teams, really recognise that, you know, we’re aware that you’re busy. We’re aware that you are stretched, and we want to be considerate about that.’* (*Researcher ID 2).*

*‘But when you go into a team meeting, there’s… you’re the experts here, how can we get your experience, you know, whatever works for you. I’m just a researcher, what do I know?...... I’ve definitely sensed some people coming in with this kind of like what now? And just kind of start off by apologising for your very existence and then you get a bit of a thaw…. after they’ve given you a bit of a mauling you get some other people in the team who will then kind of feel a bit bad.’* (*Researcher ID 6).*

##### Theme B7: Navigating through the clinical world (*facilitator*)

Researchers reported that they had to quickly work out who held the power within a clinical team and how a difference of views between team members could impact on how they worked with clinicians.

*‘Because the psychiatrist is all on board, but the psychologist isn’t and then it’s just like oh who do we listen to…yeah sometimes it can be a bit tense.’ (Researcher ID5).*

‘*Very, very quickly in the team meeting it became apparent that the admin staff was key to this entire process. There was a single person to get on board and once they were enthusiastic about it, everything was fine’* (*Researcher ID 6).*

Researchers discussed the use of ‘soft’ skills to build and maintain relationships with clinical staff. People with clinical experiences who had become researchers were able to use some of their transferable skills.

*“It’s a lot about the soft skills, the relationships with people. Often when talking about research it’s the language that I use…I think looking at a longer-term relationship, having a very high threshold, being really understanding has been beneficial to work with people for a longer period of time” (Dual ID8).*

*‘And just being present, you know, every other week, being a visible force and just smiling and talking to people and just trying to be nice……I think I was really, really helped in that because I am a clinician and because I could read what was going on. I could understand that, you know, what sounded like a kind of huge drama, I think, actually I know this is all going to die down in five minutes or… And I could sort of, I could second guess what was going to happen’* (*Dual ID7).*

##### Theme B8: The demands of the researcher role (*barrier*)

Researchers felt pressured to meet recruitment targets and worked hard to find ways to achieve this, whilst trying to be respectful of the demands on clinician’s time.

*‘The very nature of the research is that we have recruitment targets, we need to keep going, we need these numbers, so you kind of just start to… use your initiative and come up with new ideas. You always have to be thinking oh that didn’t work, what else can I do?’ (Researcher ID2).*

*‘Some of the ways we try to get practitioners to help us is, you know, lots of buttering up, you know, presents, cards, Nandos, you know, it got ridiculous sometimes’* (*Dual ID9).*

Most researchers also reported that learning how to engage clinicians in research and work collaboratively was done without any formal training. They stated they learned by trial and error and coped with difficult situations by being resilient. Whereas those in a dual role used their transferable skills.

*(I learned through) ‘Trial and error…..getting it wrong’ (Researcher ID3). ‘I learned on the job’* (Researcher ID1).

*‘I think one of the ways I feel like I’ve used my experiences as a healthcare professional is a lot of that about persuading people to do things that they don’t really want to do’ (Dual ID7).*

*‘Having a sort of thick skin about not being initially put off … it’s kind of knowing that you can sometimes push a bit harder to get there or, you know, change tack’ (Dual ID7).*

Another challenge for researchers was working across the academic and clinical worlds and having to deal with the differences in expectations from the people in both organisations.

*‘People outside research don’t see how intensely wracked with doubt most researchers are about absolutely everything…, there’s lots and lots of training that you go on and you do presentations and you’re told to look confident ….And I think that confidence in a clinical setting when you’re talking to people you don’t know sometimes can come across as arrogance and talking down to people and trying to sound clever’ (Researcher ID6).*

*‘I think there are some people who really have this sort of fixed on idea that you are some sort of ivory tower, that you have absolutely no conception of the real world, like whatsoever. And that can be difficult to break down.’ (Researcher ID3).*

## Discussion

The findings from our focus groups with 18 participants working in clinical and/or research roles indicate there was agreement within and between focus groups about the barriers that impede engagement in and with research and the facilitators that enable collaborative working relationships. There was also agreement between clinician and researcher participants that the responsibility for the forming and maintenance of collaborative and positive relationships lie with the researcher. In all groups there were no dissenting views expressed. The issues identified were agreed upon in each focus group.

Clinician participants in our study expected researchers to be able to demonstrate that they understood why and how their research study would be of benefit to service users and clinicians. If the research study was not perceived as beneficial or was perceived to be done for other reasons (e.g. for researchers to publish a paper and advance their own career), clinicians would be less likely to engage with the research. The clinician’s perceptions of the researcher’s interpersonal qualities and communication skills negatively or positively influenced their engagement with the research. Being able to sell the study, being energetic and charismatic and being empathic to the challenges of clinical work were favoured by clinician participants. Not being kept up to date on the progress or results of research projects they were engaged in was demoralising and led to clinicians being wary of being involved in future projects.

The views of the clinician participants in our study are similar to those reported in previous studies. A meta narrative review of enablers and barriers of the initiation of research and research user partnerships [11] identified that the personality of the researcher was seen as an enabler to integrated knowledge transfer when perceived as positive and as a barrier when perceived as negative by research users. Attitudes about researchers and the perceived value of research were also identified as being important in enabling or getting in the way of initiating research in health care settings [11].

Clinician participants also reported not having protected time to engage in research and reported a lack of organisational support. The majority of clinician participants wanted to be more involved in research studies but were unable to get involved to the extent they would like to. This finding is supported by other research that suggests that clinicians want to undertake research, but the majority do not have protected time and if they do, the opportunity is often lost because of competing clinical demands [11, 28–30]. Clinicians in our study were frustrated they were not involved in the development of research studies and are usually first aware of a particular study when they are asked to recruit participants from their caseload. This suggests that collaboration between some of the clinicians in our study and the researchers they had worked with related to engaging with research rather than in research [10]. Researcher participants in our study did not raise the issue of the need for clinicians to be involved earlier in the research process, though this has been highlighted in other studies. Findings from a survey of clinical studies officers (CSOs) employed by the Mental Health Research Network, (an NIHR network that facilitates research in the NHS) reported that building good relationships with clinicians was key to successful recruitment and suggested involving clinicians at an early stage in the research process [31].

Researcher participants demonstrated qualities that clinicians said they valued and were needed to engage them in a research project. All researcher participants in our study, regardless of whether they had prior clinical experience, demonstrated they understood the demands of the clinical world and were respectful of the demands on clinician’s time. They also acknowledged that it was their role to ensure successful working relationships were built and maintained. The importance of researchers building and maintaining good relationships to help support the research process participant is illustrated by the work of Peckham et al. regarding the SCIMITAR+ study, a multi-centre randomised controlled trial of a smoking cessation intervention for people with serious mental illness [32, 33]. The research team worked closely with clinicians in 21 community mental health and 16 primary care sites in the UK. In some sites, researchers were embedded into clinical teams which allowed researchers to understand how best to recruit in each team, and their continued presence during the trial meant that clinical staff had someone familiar as a point of contact. Peckham et al. [32] reported that the main factors that led to successful recruitment and retention was the relationship between researchers and the recruiting clinicians, as well as the recruiting clinicians and study participants.

Clinician participants and those with a dual role perceived that researchers with a clinical background may be better prepared to work collaboratively with clinicians due to their prior knowledge and experience of clinical settings. Other research supports this idea e.g. [13]. However, the researchers without a clinical background in our study demonstrated they understood the demands of the clinical world and had eventually learned to navigate it.

Research so far has mostly focused on how clinicians or users of research perceive researchers or the research process and how to support clinicians to be more involved in research [34, 35]. There has been less of a focus on how to support researchers (who are often junior research assistants on temporary contracts for the duration of the study) to conduct research in clinicals settings. Researcher participants in our study described learning on the job and needing to be ‘thick skinned’ and resilient to meet the demands of their job roles. Patterson et al. [36] conducted workshops with 19 experienced researchers to build a framework to support successful trial recruitment. They reported that researchers needed to have ‘ingenuity and persistence’ to initiate and maintain relationships with clinicians and that personality, interpersonal skills and credibility of researchers are important. The study also reported that researchers need to be emotionally resilient to repeated rebuffs and outright rejection from clinicians and research participants. Borschmann et al. [31] also highlighted the importance of having access to practical and emotional support from supervisors and colleagues, in addition to access to training.

Our findings, which are based on a small study in one NHS Trust and one University, have some implications for partnership working within our ARC going forward, and also may have wider applicability for mutually beneficial programmes of implementation research and attempts to bridge the second translational gap. First, NHS organisations and Universities need to continue to develop strategies for allowing clinical staff to have the time to be involved in research in a meaningful way, so that clinicians are not left feeling they are compromising their clinical work. Clinicians perceived lack of time and heavy workloads should not be a barrier to research and their universities involving clinicians (and service users) in the early developmental and design stages of research studies to ensure that research is seen as beneficial to clinical staff and service users. Secondly, developing capacity to have an indepth understanding of each partner’s role so that expectations of each other can be managed effectively would be helpful. This may involve new ways of working including using the ‘Embedded Researcher’ or Researcher-in-Residence model where a researcher works within clinical services and works directly with clinical staff on understanding what research they would like to see done and supporting them to be more involved in designing and running research projects [37, 38]. Thirdly, there needs to be more training and support for researchers on how to work best with clinicians. This could include ensuring researchers understand the clinical area they are working in, the challenges of clinical work and how to be a good ally to clinical staff. This could be delivered by clinicians or staff with a dual clinician/researcher role. Future research studies could evaluate different models of research and knowledge transfer using RCTs and qualitative studies to understand the mechanisms involved and which relationships work best.

### Strengths and limitations

We were able to draw on our own clinical and research experience in designing the interview guide, conducting the focus group discussions and interpreting the data. The NHS Trust and University we recruited from have very close links and the staff who work in these organisations have shared experiences. However, there are several limitations which may have affected the rigor of the research. These settings may not be representative of other mental health organisations in the UK. SLaM is the most research active mental health Trust in the UK [39] and clinicians (and service users) are in high demand to participate in research. One the one hand this means that they are exposed to novel health innovations, however, as our participants indicated this also leads to research fatigue, something which many clinicians in the UK who rarely get to participate in research, may not be able to relate to. Similarly, study participants and the experiences they discussed may not be representative of the local or wider workforce. We appeared to recruit a group of motivated clinicians who were interested and engaged in research and we failed to recruit and elicit views of those less interested and detached from research. There are difficulties in recruiting clinical staff who are not interested in research in research studies, as by their very lack of interest in research they are less likely to agree to take part in a research study. It is therefore possible we may not have reached data saturation about this topic. This may impact on the credibility of our findings as it may be that there is more that we could have learnt about clinical staff and researcher views on working together on research These limitations which may affect the rigor of the research findings, could be overcome by future studies including participants from more than one clinical and academic organisation and from different geographical areas. Efforts to include staff less engaged with research, using additional research designs e.g. anonymised surveys to triangulate the data may help. Further member checks with participants and not just between participants could have been carried out and may have resulted in participants providing different interpretations of the findings, however the usefulness of member checking in qualitive research has been questioned [40].

## Conclusions

Building and maintaining good working relationships with clinicians is a key task for researchers. There is a breadth of research outlining what clinicians want from researchers and their organisation to enable them to be more involved in research, this includes protected time to work in partnership with researchers throughout the life cycle of a research project. Whereas, supporting researchers in their role calls for training in communication skills and bespoke training about understanding the local context in which research is taking place. More thought needs to be given to the process of building relationships as well as the technical aspects of managing research studies, particularly large randomised controlled trials. Research organisations and senior staff need to support researchers with this important task.

## Supplementary Information

**Additional file 1.**

## Data Availability

The data from the focus groups are not available to ensure the anonymity of participants.

## Abbreviations

NIHR: National Institute for Health Research
NHS: National Health Service
CLAHRC: Collaborations for Leadership in Applied Health Research and Care
ARC: Applied Research Collaboratives
SLaM: South London and Maudsley NHS Trust
IoPPN: Institute of Psychiatry, Psychology and Neuroscience
KCL: King’s College London
COREQ: Consolidated criteria for reporting qualitative research
