# Supplementary material for: Barriers and facilitators of clinician and researcher collaborations: a qualitative study
Source: BMC Health Serv Res. 2020 Dec 5;20:1126. doi: 10.1186/s12913-020-05978-w (PMC7718701; doi:10.1186/s12913-020-05978-w)
Supplement: Supplementary file 1 — Additional file 1. [file 12913_2020_5978_MOESM1_ESM.docx]

**Interview topic guide**

For all participants:

Introduce facilitators, purpose of focus group, ground rules, participants introduce themselves

Clinicians

The focus of the group is to elicit your views about……

- Your experience of working with researchers – in the context of any current and previous research projects
- Your relationship with researchers 

  Prompts

  Tell us about your experience of working with researchers in the context of any current and previous research projects, e.g.
  - Explaining the purpose of the research to you
  - Them asking you to help recruit service users/carers
  - Working with researchers to implement research and evidence based practice
  - What have researchers done that was helpful to engage you in participating in research
- Give examples of good working relationships and not so good working relationships with researchers, eg
  - The knowledge, attitudes, skills, personal style of the clinician/team
  - Your own personal style

Researchers

The focus of the group is to elicit your views about……

- Your experience of working with clinicians  – in the context of any current and previous research projects
- Your relationship with clinicians 

  Prompts 

  Tell us about your experience of working with clinicians in the context of any current and previous research projects, e.g.
  - Explaining the purpose of the research to them
  - Asking to help recruit service users/carers
  - Working with clinicians to implement research and evidence based practice
  - What helped you engage clinicians to participate in research
- Give examples of good working relationships and not so good working relationships with clinicians, eg
  - The knowledge, attitudes, skills, personal style of the clinician/team
  - Your own personal style
